# Early Open-Lung Ventilation Improves Clinical Outcomes in Patients with Left Cardiac Dysfunction Undergoing Off-Pump Coronary Artery Bypass: a Randomized Controlled Trial

**DOI:** 10.5935/1678-9741.20160057

**Published:** 2016

**Authors:** Douglas W. Bolzan, Walter José Gomes, Isadora S. Rocco, Marcela Viceconte, Mara L. S. Nasrala, Hayanne O. Pauletti, Rita Simone L. Moreira, Nelson A. Hossne Jr, Ross Arena, Solange Guizilini

**Affiliations:** 1 Disciplina de Cirurgia Cardiovascular e Cardiologia da Escola Paulista de Medicina da Universidade de São Paulo (EPM-UNIFESP), São Paulo, SP, Brazil.; 2 Departamento de Ciência do Movimento Humana, Escola de Fisioterapia da Universidade Federal de São Paulo (UNIFESP), Santos, SP, Brazil.; 3 Department of Physical Therapy and Integrative Physiology Laboratory, College of Applied Health Sciences, University of Illinois at Chicago, Chicago, USA.

**Keywords:** Coronary Artery Bypass, Off-Pump, Respiration, Artificial, Positive-Pressure Respiration

## Abstract

**Objective:**

To compare pulmonary function, functional capacity and clinical outcomes amongst three groups of patients with left ventricular dysfunction following off-pump coronary artery bypass, namely: 1) conventional mechanical ventilation (CMV); 2) late open lung strategy (L-OLS); and 3) early open lung strategy (E-OLS).

**Methods:**

Sixty-one patients were randomized into 3 groups: 1) CMV (n=21); 2) L-OLS (n=20) initiated after intensive care unit arrival; and 3) E-OLS (n=20) initiated after intubation. Spirometry was performed at bedside on preoperative and postoperative days (PODs) 1, 3, and 5. Partial pressure of arterial oxygen (PaO_2_) and pulmonary shunt fraction were evaluated preoperatively and on POD1. The 6-minute walk test was applied on the day before the operation and on POD5.

**Results:**

Both the open lung groups demonstrated higher forced vital capacity and forced expiratory volume in 1 second on PODs 1, 3 and 5 when compared to the CMV group (*P*<0.05). The 6-minute walk test distance was more preserved, shunt fraction was lower, and PaO_2_ was higher in both open-lung groups (*P*<0.05). Open-lung groups had shorter intubation time and hospital stay and also fewer respiratory events (*P*<0.05). Key measures were significantly more favorable in the E-OLS group compared to the L-OLS group.

**Conclusion:**

Both OLSs (L-OLS and E-OLS) were able to promote higher preservation of pulmonary function, greater recovery of functional capacity and better clinical outcomes following off-pump coronary artery bypass when compared to conventional mechanical ventilation. However, in this group of patients with reduced left ventricular function, initiation of the OLS intra-operatively was found to be more beneficial and optimal when compared to OLS initiation after intensive care unit arrival.

**Table:** 

Abbreviations, acronyms & symbols				
**6MWT**	**=Six-minute walk test**		**L-OLS**	**=Late open lung ventilation strategy**
**CMV**	**=Conventional mechanical ventilation**		**OLS**	**=Open lung ventilation strategy**
**E-OLS**	**=Early open lung ventilation strategy**		**OPCAB**	**=Off-pump coronary artery bypass**
**FEV_1_**	**=Forced expiratory volume in 1 second**		**PaCO_2_**	**=Partial pressure of arterial carbon dioxide**
**FiO_2_**	**=Fraction of inspired oxygen**		**PaO_2_**	**=Partial pressure of arterial oxygen**
**FVC**	**=Forced vital capacity**		**PEEP**	**=Positive end-expiratory pressure**
**ICU**	**=Intensive care unit**		**POD**	**=Postoperative day**

## INTRODUCTION

Open lung ventilation strategy (OLS) consists of the application of short periods of high inspiratory pressures (to open the collapsed alveoli) associated with a relatively high level of positive end-expiratory pressure (PEEP) - to keep the alveoli open - and low tidal volumes^[1]^. Recently, this strategy has demonstrated to be efficient in promoting faster improvement in functional and clinical parameters (*i.e.*, pulmonary function, functional capacity, and clinical outcomes) following off-pump coronary artery bypass grafting (OPCAB) when compared to conventional mechanical ventilation (CMV)^[1]^. In this previous study, no differences were found between the OLS initiated intra-operatively and after intensive care unit (ICU) arrival. Nevertheless, the study included only patients with preserved left ventricular function.

Future investigations assessing OLS interventions focused on patients with left ventricular dysfunction undergoing OPCAB are needed to determine whether there are instances where intraoperative initiation of this strategy would be beneficial. Therefore, the purpose of this study was to compare pulmonary function, functional capacity, and clinical outcomes amongst three groups of patients with left ventricular dysfunction following OPCAB: 1) CMV; 2) CMV plus early OLS (E-OLS); and 3) CMV plus late OLS (L-OLS).

## METHODS

This study was conducted at Universidade Federal de São Paulo, São Paulo, Brazil. The Human Ethics Research Committee of our institution approved this study and every patient gave informed written consent. We prospectively studied 61 patients with stable obstructive coronary artery disease and ventricular dysfunction (left ventricular ejection fraction ≤45%), who electively underwent first-time OPCAB between January 2007 and June 2012. Patients were excluded if: 1) were submitted to emergency CABG or reoperation; 2) presented with acute or chronic pulmonary disease; 3) presented with an intraoperative event (*e.g*., pulmonary edema, arrhythmias, and cardiac arrest); or 4) required prolonged time of CMV (>24 hours). Randomization was undertaken by a computer software and the patients were allocated into three groups: 1) CMV (n=21); 2) L-OLS (n=20); and 3) E-OLS (n=20). We used numbered, sealed and opaque envelopes to guarantee confidentiality. Pulmonary function and clinical outcomes served as primary endpoints. Functional capacity was our secondary endpoint.

### Endpoints

The endpoints and the operative technique in this study followed the standard employed in our former report^[1]^.

Pulmonary function was evaluated at the bedside by forced vital capacity (FVC) and forced expiratory volume in 1 second (FEV_1_), on the day before surgery and on postoperative days (POD) 1, 3, and 5, by the same physiotherapist blinded to the patient's group allocation. The evaluation was performed by a portable spirometer (Spirobank G, MIR, Rome, Italy), in accordance with the American Thoracic Society guidelines^[2]^.

Arterial blood samples were collected for the analysis of partial pressure of arterial oxygen (PaO_2_) and partial pressure of arterial carbon dioxide (PaCO_2_) on the preoperative period and on POD1, always before spirometry with the patient breathing room air. Pulmonary shunt fraction was calculated using the Oxygen Status Algorithm software (Version 2.0; Mads&Ole Siggaard; Radiometer).

The six-minute walk test (6MWT) was applied for evaluation of submaximal functional capacity. An experienced physiotherapist, blinded to the patient's group assignment, conducted the 6MWT on the day before surgery and on POD5, in accordance with the American Thoracic Society guidelines^[3]^. For all patients, predicted 6MWT distances were preoperatively calculated according to the equation proposed by Iwama et al.^[4]^.

### Intraoperative

The same anesthetic regimen was used for all patients. Anesthesia was induced with etomidate and midazolam and maintained with sufentanil and isoflurane (0.5% to 1%). According to our previous randomization, after the intubation procedure, the patients were allocated to one of the three groups, and ventilated by the same mechanical ventilator (Takaoka, Brazil):

- **Conventional mechanical ventilation group (n=21):** These patients were used as our control group. CMV was started with volume control ventilation immediately after intubation and the following settings were adopted: 1) tidal volume of 8 mL/kg of predicted body weight; 2) 0 cmH_2_O PEEP, inspiration/expiration ratio of 1:2; 3) fraction of inspired oxygen (FiO_2_) set to guarantee a PaO_2_ between 80 and 100 mmHg; and 3) respiratory rate adjusted to keep PaCO_2_ between 35 and 45 mmHg. After surgery, the patients were transported to the ICU using a portable mechanical ventilator (Takaoka, Brazil), maintaining the same ventilatory settings, but with PEEP at 5 cmH_2_O. After ICU arrival, ventilation continued with PEEP at 5 cmH_2_O and the other aforementioned settings were maintained until the weaning started.
- **Late open lung strategy group (n=20):** The same parameters used in the CMV group were followed after intubation, during surgery, and during transport to the ICU. Thirty minutes after ICU arrival, CMV was changed to an OLS, which was maintained until extubation. The OLS was initiated in pressure-controlled ventilation mode with the following settings: 1) FiO_2_ to keep the PaO_2_ between 80 and 100 mmHg, 10 cmH_2_O PEEP; 2) inspiration/expiration ratio of 1:2; 3) inspiratory pressure to achieve a tidal volume of 6 mL/kg of predicted body weight; and 4) respiratory rate adjusted to maintain a PaCO_2_ between 35 and 45 mmHg. Additionally, as part of the OLS, a lung re-expansion maneuver was employed by increasing peak inspiratory pressure to 40 cmH_2_O for 15 seconds to keep the PaO_2_/FiO_2_ ratio higher than 375 mmHg. If the PaO_2_/FiO_2_ ratio decreased below 375 mmHg due to an accidental disconnection, a new re-expansion maneuver was implemented. The OLS was maintained until the weaning procedure started.
- **Early open lung strategy group (n=20):** These patients underwent pressure-controlled ventilation with OLS as previously described, but starting immediately after intubation. Ventilation according to the OLS was kept during the entire operative period and in the ICU. During surgery, whenever lung expansion blocked surgical exposure, PEEP was decreased to 5 cmH_2_O to construct the distal grafts, and lung deflation time was documented. We restored the ventilation settings used before the lung deflation as soon as possible. In addition, a lung re-expansion maneuver was performed by increasing peak inspiratory pressure to 40 cm H_2_O for 15 seconds to achieve a PaO_2_/FiO_2_ ratio greater than 375 mmHg. After surgery, the patients were transferred to the ICU using a portable mechanical ventilator (Takaoka, Brazil), and the same ventilatory settings were maintained. Ventilation with an OLS as previously described was kept until weaning initiation.

### Operative Technique and Postoperative

The procedure was carried out according to our standardized protocol^[5]^. During surgery, temperature and preload were continuously monitored and a heated water mattress was used to maintain all patients normothermic. The OPCAB surgery was performed through a median sternotomy. The left internal thoracic artery was harvested by skeletonized technique and complemented with additional saphenous vein grafts. OPCAB followed our standards, with systemic heparinization to achieve an activated clotting time of over 250 seconds; proximal soft silicone snare was used for temporary occlusion of the grafted coronary artery. Distal anastomosis was accomplished with a 7-0 running polypropylene suture and stabilization was achieved with an Octopus 3 (Medtronic, Inc, Minneapolis, MN, USA), which was used in all cases. The vein top ends were connected to the ascending aorta using tangential clamping.

After surgery, the patients were transferred to the ICU and ventilated according to CMV or OLS protocols, determined by previous randomization. When the patient was able to trigger the ventilator, we changed the ventilatory mode to pressure support ventilation. To compensate for the circuit and endotracheal tube resistance, a support level of 7 cmH_2_O was adopted. PEEP was adjusted to 10 cmH_2_O in both OLS groups and it remained unchanged during weaning until complete extubation. We kept PEEP levels in the CMV group at 5 cmH_2_O. Extubation was done when the patient was alert to maintain self-ventilation and had good blood gas values as well as hemodynamic stability. During the first 5 POD all patients received the same analgesia regimen (tramadol chlorhydrate - 100 mg four times a day). Pain sensation was evaluated on POD1, always before spirometry, and quantified by a modified standard score (0=no pain to 10=unbearable pain). The drains (mediastinal and/or pleural) were routinely removed on POD2. On the postoperative period, the patients were guided by a physiotherapist to perform breathing exercises until discharge.

### Clinical Outcomes

Time on assisted ventilation, postoperative in-hospital length of stay, and respiratory events (pleural effusion, pneumothorax, atelectasis, and pneumonia) were documented according to our previous methodology^[1]^. Chest roentgenograms, obtained preoperatively and on POD1, 3 and 5, were evaluated by a radiologist blinded to the patient's group allocation.

### Statistical Analysis

Data are reported as mean ± standard deviation. Based on a previous study^[6]^, the sample size calculation considered FVC at POD1, taking into account a significance level of 5% and 80% power to detect a difference between groups of at least a 400 ml decrease compared to the preoperative period. This assumption suggested a sample of 60 patients, resulting in a total of 75 patients to account for subjects not completing the study. The values of FVC, FEV_1_ and 6MWT distance were expressed as a percentage of the preoperative value. Within-group variables comparing preoperative *versus* postoperative values were evaluated by paired Student t test and repeated measures ANOVA. The unpaired Student t test and the Mann-Whitney test for comparison between groups were used when necessary. The categorical variables were analyzed by the Pearson Chi-square test. A *P*-value <0.05 was considered statistically significant for all tests. Statistical analyses were performed using GraphPad Prism 3.0 Software (GraphPad Software Inc, San Diego, CA, USA).

## RESULTS

**Fig. 1:**
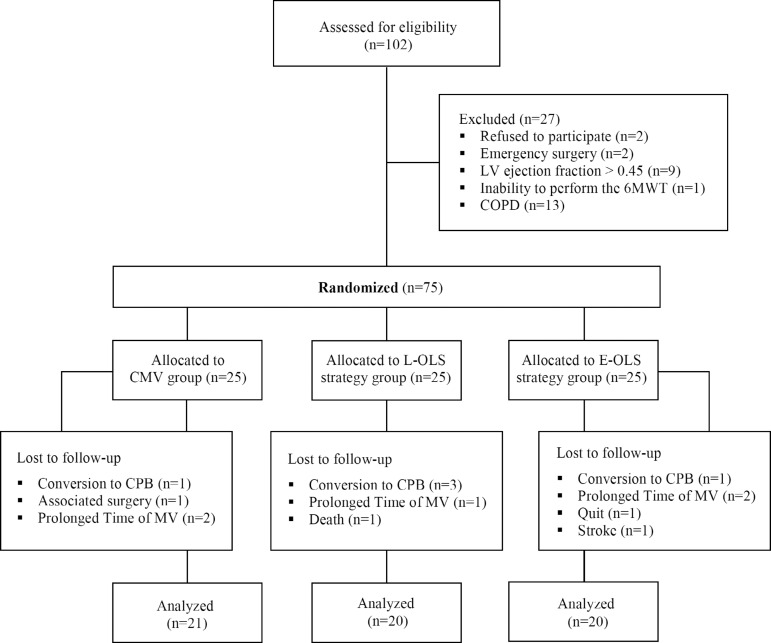
Participants flow. 6MWT=six-minute walk test; CMV=conventional mechanical ventilation; COPD=chronic obstructive pulmonary disease; CPB=cardiopulmonary bypass; E-OLS=early open lung ventilation strategy; L-OLS=late open lung ventilation strategy; LV=left ventricular; MV=mechanical ventilation

During the study period, 102 patients were assessed for eligibility and, from that sample, 61 were in fact analyzed (Figure 1). The groups were homogenous in terms of perioperative characteristics (Table 1).

**Table 1:** Pre- and intraoperative clinical and demographic parameters.

Variables	CMV Group (n=21)	E-OLS Group (n=20)
Age (years)	58.54±14.32	59.43±13.68
Male/female (n)	15/6	15/5
BMI (kg/m^2^)	25.42±1.22	25.89±2.41
LVEF	0.39±0.04	0.40±0.03
EuroSCORE II	6.4±2.2	6.6±3.4
**Pulmonary function**		
FVC (L)	3.21±0.44	3.35±0.54
% predicted	84.13±14.26	84.86±16.12
FEV_1_ (L)	2.64±0.42	2.71±0.48
% predicted	92.21±13.45	89.88±14.85
PaO_2_ (mmHg)	78.26±5.13	78.83±3.96
PaCO_2_ (mmHg)	38.49±3.12	39.28±3.83
Shunt fraction (%)	0.04±0.01	0.03±0.01
6MWT (m)	385.6±18.88	389.2±25.23
Operative time (min)	310.4±22.84	316.2±24.48
Intact pleura (n)	11	9
Grafts per patient (n)	2.48±0.47	2.56±0.44
IO fluid balance (mL)	2,730±438	2,780±360

Data shown as mean ± standard deviation BMI=body mass index; CMV=conventional mechanical ventilation; E-OLS=early open lung ventilation strategy; FEV_1_=forced expiratory volume in 1 second; FVC=forced vital capacity; L-OLS=late open lung ventilation strategy; LVEF=left ventricular ejection fraction; PaCO_2_ =partial pressure of arterial carbon dioxide; PaO_2_=partial pressure of arterial oxygen; 6MWT=six-minute walk test; IO=Intraoperative *P<0.05

Regardless of the mechanical ventilation strategy, all groups demonstrated a significant impairment in pulmonary functional parameters (FVC and FEV_1_) until POD5, in comparison with preoperative values (*P*<0.05). However, the E-OLS group showed higher FVC and FEV_1_, expressed as a percentage of the preoperative value, on PODs 1, 3 and 5, compared to the L-OLS and CMV groups. When the L-OLS and CMV groups were compared to each other, pulmonary function variables were significantly higher in the L-OLS group in comparison with the CMV group (Table 2).

**Table 2:** Pulmonary function test values on PODs 1, 3 and 5, in percentage of the preoperative values.

	CMV Group	L-OLS Group	E-OLS Group	
	FVC (%)	FVC (%)	FVC (%)	FEV_1_ (%)
POD1	32.22±9.4^a**^	40.55±10.43^c*^	48.65±11.2^b**^	49.44±13.8^b**^
POD3	39.54±12.2^a*^	51.33±11.02^c*^	67.33±13.27^b**^	65.22±11.9^b**^
POD5	65.67±14.9^a**^	77.45±15.66^c*^	89.33±15.20^b**^	86.51±14.3^b**^

Data shown as mean ± standard deviation CMV=conventional mechanical ventilation; E-OLS=early open lung ventilation strategy; FEV_1_=forced expiratory volume in 1 second; FVC=forced vital capacity; L-OLS=late open lung ventilation strategy; POD=Postoperative day Comparison between groups:

a CMV *vs*. L-OLS;

b CMV *vs*. E-OLS; and

c L-OLS *vs*. E-OLS

* *P*<0.05;

** *P*<0.001

Similar results were found when other variables were compared. A significant increase in pulmonary shunt fractions and a drop in PaO_2_ at POD1 were observed in all groups in relation to preoperative data (*P*<0.05). However, the E-OLS group showed higher values of PaO_2_ and reduced pulmonary shunt fractions when compared to the L-OLS and CMV groups. A significant difference was observed in terms of the aforementioned variables when the L-OLS and CMV groups were compared. The L-OLS group presented higher values of PaO_2_ and decreased pulmonary shunt fractions compared to the CMV group. The PaCO_2_ values increased in the three groups and similar results were found amongst them (Table 3).

**Table 3:** Arterial blood gas and shunt fraction measurements in the first postoperative day.

Variables	CMV Group	E-OLS Group
PaO_2_(mmHg)	58.72±1.86^a*^	75.18±2.46^b*^
PaCO_2_(mmHg)	39.27±2.13^a^	38.12±1.58^b^
Shunt fraction (%)	0.27±0.01^a*^	0.13±0.01^b*^

Data expressed as mean ± standard deviation CMV=conventional mechanical ventilation; E-OLS=early open lung ventilation strategy; L-OLS=late open lung ventilation strategy; PaCO_2_=partial pressure of arterial carbon dioxide; PaO_2_=partial pressure of arterial oxygen

* *P*<0.0001 refers to comparison between groups:

a CMV *vs*. L-OLS;

b CMV *vs*. E-OLS; and

c = L-OLS *vs*. E-OLS

On POD5, a significant decrease was observed for the 6MWT distance in all groups (*P*<0.05). However, the distance (expressed as percentage of the preoperative value) was significantly higher in the E-OLS group (81.13±1.67%) when compared to the L-OLS (74.87±1.49%) and CMV group (68.12±2.09%); *P*<0.001. When the distance covered by the L-OLS and CMV groups was compared to each other, the L-OLS group presented a higher significant difference in relation to the CMV group (*P*<0.05).

Adverse postoperative respiratory events (atelectasis, pleural effusion and pneumonia) were higher in the CMV group compared to both OLS groups until POD5. When the L-OLS and E-OLS groups were compared to each other, a significantly higher prevalence of atelectasis and pneumonia was observed in the L-OLS group (Table 4). No cases of pneumothorax were observed in any of the three groups.

**Table 4:** Postoperative clinical outcomes.

Variables	CMV Group (n=33)	L-OLS Group (n=31)	E-OLS Group (n=31)
Atelectasis, n (%)	14(42.42)^a*^	8(25.80)^c*^	4(12.40)^b*^
Pleural effusion, n (%)	9(27.27)^a*^	5(16.12)^c*^	3(9.67)^b*^
Pneumonia, n (%)	8(24.24)^a*^	4(12.90)^c*^	1(3.22)^b*^
Mechanical ventilation time, hours**	14.56±2.26^a*^	12.22±1.48^c*^	9.49±1.47^b*^
Hospital stay, days**	10.89±1.83^a*^	8.42±1.74^c*^	6.88±1.45^b*^

** Data shown as mean ± standard deviation CMV=conventional mechanical ventilation; E-OLS=early open lung ventilation strategy; L OLS=late open lung ventilation strategy Comparison between groups:

a CMV *vs*. L-OLS;

b CMV *vs*. E-OLS; and

c L-OLS *vs*. E-OLS.

* *P*<0.05

Mechanical ventilation time and length of hospital stay after OPCAB was shorter in the E-OLS group in comparison to the other ones. However, the CMV group presented with a significantly longer time of intubation and hospitalization in comparison to the L-OLS group (Table 4).

During application of the E-OLS, lung inflation impaired surgical exposure in 6 patients, necessitating the decrease of PEEP from 10 to 5 cmH_2_O to construct the distal grafts, resulting in a mean deflation time of 8.7±1.1 minutes.

## DISCUSSION

In the present study, the OLS demonstrated better preservation of pulmonary function and recovery, functional capacity recovery, and clinical outcomes when compared to CMV in patients with left ventricular dysfunction who underwent OPCAB, especially when the OLS was applied earlier. Similar results were found in a study by Miranda et al.^[7]^. However, in this previous study^[7]^, the patients had preserved left ventricular ejection fraction, which is a key characteristic differing from patients included in the current study. According to the literature assessing the on-pump surgical technique, the early reperfusion period is characterized by a high incidence of regional or global ventricular dysfunction, even in patients with a normal preoperative ejection fraction^[8,9]^. Perhaps this fact has contributed to the similarity of results in the present and previous investigations, despite the differences existent in terms of cardiac function amongst the aforementioned studies. In addition, evidence suggests that cardiopulmonary bypass produces greater impairment in lung function when compared to OPCAB in the early postoperative period^[10-12]^. OPCAB has been demonstrated to afford better outcomes in patients with left ventricular dysfunction compared to the on-pump technique^[13]^.

To our knowledge, this is the first study comparing the aforementioned outcomes between OLS and CMV in patients with left cardiac dysfunction who underwent OPCAB. Previous investigations demonstrated that OLS is more effective in preserving key clinical parameters and/or outcomes when compared to CMV in patients with preserved cardiac function^[1]^. Therefore, the novel aspect of the current study is the evaluation of the OLS on pulmonary function, functional capacity, and clinical outcomes in a population with impaired left ventricular function.

In our previous investigation^[1]^, unlike what was observed in the present study, no differences were found in relation to pulmonary function (FVC and FEV_1_) when E-OLS and L-OLS were compared. We believe that, in the previous study, patients with preserved cardiac function may have counterbalanced the aggressive fluid infusion needed to maintain hemodynamic stabilization and allow for heart displacement to construct the distal grafts, thereby preserving the lungs. Moreover, the lower negative impact on spirometric data in the E-OLS group observed in this study may be related to the relatively high level of PEEP (10 cm H_2_O) that kept the alveoli open and possibly promoted a more effective fluid distribution during ventilation in the intraoperative period. This mechanism may explain other findings, in particular, the group of patients with better pulmonary function preservation also presented with better oxygenation and lower pulmonary shunt fractions. Previous evidence suggests that PEEP could improve oxygenation during the early postoperative phase of cardiac surgery^[14-18]^. We hypothesize that this improvement in gas exchange could be associated with the reexpansion maneuvers (for pulmonary opening) associated with PEEP, which maintained the alveoli open.

Our previous investigation^[1]^ also demonstrated an association between pulmonary function impairment and greater loss in functional capacity. This association was also observed in the present study; the patients with poorer pulmonary function (*e.g*., CMV and L-OLS groups) also had shorter 6MWT distances.

According to previous research, due to the multifactorial impairment of pulmonary function^[5,19-23]^, there is a significant risk of respiratory complications during the postoperative period, which may result in prolonged time of mechanical ventilation, longer postoperative hospital stay, and possibly death^[5,20,21]^. Those results are in agreement with our findings; the CMV and L-OLS groups with greater pulmonary function impairment also showed a significantly greater intubation time and hospital stay in comparison to the E-OLS group.

### Clinical Implications

In the present study, the OLS intervention was responsible for better preservation of respiratory and functional capacity, lower prevalence of respiratory events and reduction in hospital stay in patients with left ventricular dysfunction undergoing OPCAB. We also observed that the earlier we begin the OLS in this profile of patients, the greater the beneficial effects are. These findings may allow for the stratification of patients regarding ventilatory strategies to be adopted, possibly having a positive impact on the patient's ability to perform activities of daily living upon discharge.

### Study Limitations

Technical difficulties were found in performing the grafts in 6 patients when a 10 cmH_2_O PEEP was used due to surgical exposure obstruction by the hyperinflated lungs. In those cases, PEEP was decreased to 5 cmH_2_O. However, PEEP was reduced for graft construction for a small period of time (approximately 9 minutes); a reexpansion maneuver was applied to minimize this derecruitment and PEEP was restored soon as possible. Thus, we believe that those maneuvers did not significantly influence our study findings.

## CONCLUSION

Both OLSs (L-OLS and E-OLS) were able to promote higher preservation of pulmonary function, greater recovery of functional capacity, and better clinical outcomes following OPCAB when compared to CMV. However, in this group of patients with reduced left ventricular function, initiation of the OLS intra-operatively was found to be more beneficial and optimal when compared to OLS initiation after ICU arrival.

**Table:** 

Authors’ roles & responsibilities	
DWB	Conception and design study; manuscript redaction or critical review of its content; final manuscript approval
WJG	Conception and design study; final manuscript approval
ISR	Analysis and/or data interpretation; manuscript redaction or critical review of its content; final manuscript approval
MV	Manuscript redaction or critical review of its content; final manuscript approval
MLSN	Manuscript redaction or critical review of its content; final manuscript approval
HOP	Manuscript redaction or critical review of its content; final manuscript approval
RSLM	Manuscript redaction or critical review of its content; final manuscript approval
NAHJ	Manuscript redaction or critical review of its content; final manuscript approval
RA	Manuscript redaction or critical review of its content; final manuscript approval
SG	Conception and design study; manuscript redaction or critical review of its content; final manuscript approval
